# Microbial resource utilization traits and trade-offs: implications for community structure, functioning, and biogeochemical impacts at present and in the future

**DOI:** 10.3389/fmicb.2015.00254

**Published:** 2015-04-08

**Authors:** Elena Litchman, Kyle F. Edwards, Christopher A. Klausmeier

**Affiliations:** ^1^W.K. Kellogg Biological Station – Michigan State UniversityHickory Corners, MI, USA; ^2^Department of Integrative Biology, Michigan State UniversityEast Lansing, MI, USA; ^3^Department of Oceanography, University of Hawai’i at ManoaHonolulu, HI, USA; ^4^Department of Plant Biology, Michigan State University, East LansingMI, USA

**Keywords:** microbes, nutrient uptake, resource competition, diversity, trait-based models, trait evolution, global change, warming

## Abstract

Trait-based approaches provide a mechanistic framework to understand and predict the structure and functioning of microbial communities. Resource utilization traits and trade-offs are among key microbial traits that describe population dynamics and competition among microbes. Several important trade-offs have been identified for prokaryotic and eukaryotic microbial taxa that define contrasting ecological strategies and contribute to species coexistence and diversity. The shape, dimensionality, and hierarchy of trade-offs may determine coexistence patterns and need to be better characterized. Laboratory measured resource utilization traits can be used to explain temporal and spatial structure and dynamics of natural microbial communities and predict biogeochemical impacts. Global environmental change can alter microbial community composition through altering resource utilization by different microbes and, consequently, may modify biogeochemical impacts of microbes.

## Introduction

Understanding the structure, dynamics and functioning of diverse microbial communities, and their effects on biogeochemical cycles has become one of the most fast-moving and exciting areas in biology. New high throughput methods of characterizing microbial community composition have resulted in an unprecedented wealth of data that can be analyzed to make inferences about the mechanisms operating in the microbial universe. One of the promising approaches gaining momentum in microbial ecology is the trait-based framework to understand and predict community composition and dynamics. As in community ecology of macroscopic organisms such as terrestrial plants, we can use trait-based approaches to seek general patterns of community organization and identify the mechanisms structuring microbial communities. In addition, key microbial traits can give us insights into the microbial impacts on biogeochemistry and how these impacts may change in the future.

Microbes have extremely diverse metabolisms, occupy virtually all habitats on Earth and play key roles in major biogeochemical cycles. Despite all this diversity, there are some common underlying principles that govern the organization and functioning of microbial species and communities. Among those are the kinetics of resource utilization and competition for resources (Tilman, 1982; Button, 1985; Hibbing et al., 2010). There is a tremendous variety of substrates, ranging from simple inorganic ions to complex organic molecules that act as resources for different microbial groups, including the waste products of other microbes. Many of the putative resources are still poorly characterized and it is not always known what microbial groups can utilize them. However, for many resources, the Michaelis–Menten enzyme kinetics equation can be used as an adequate descriptor of microbial resource uptake (Droop, 1973; Button, 1998; Litchman and Klausmeier, 2008). Growth rate is then described by a separate equation as a function of intracellular nutrient concentration (Droop, 1973). Alternatively, the growth rate dependence on a resource can be described by a Monod equation, where growth rate is a saturating function of external resource concentration (Button, 1985; Grover, 1990). The parameters of these equations can be viewed as functional traits related to resource utilization and resource-dependent growth (Litchman and Klausmeier, 2008). These traits directly affect organism’s fitness and, therefore, are among key functional traits of organisms (Litchman et al., 2007; Violle et al., 2007).

Resource utilization traits of individual microorganisms determine their requirements for those resources and can be used to predict how a given microorganism responds to environmental conditions (resource-dependent growth). Therefore, these traits can be classified as **response traits** (Lavorel and Garnier, 2002; Suding et al., 2008). At the same time, resource utilization by microorganisms has direct effects on biogeochemical processes in ecosystems and, therefore, traits associated with resource uptake and growth are also **effect traits** (Lavorel and Garnier, 2002; Suding et al., 2008). Not all traits in microbes or macroscopic organisms can simultaneously be classified into these two categories. For example, traits characterizing growth rate responses to temperature, pH or other environmental parameters are response traits but not effect traits. Traits that are simultaneously response and effect traits tightly link the environment, microbial community structure, and biogeochemical processes and may have a higher predictive power for determining the biogeochemical impacts (Lavorel and Garnier, 2002). It would be of interest to systematically examine the relative importance of different types of traits on biogeochemistry.

Eco-physiological traits, including the resource acquisition and utilization traits, are often correlated with each other and these correlations may constitute trade-offs, if their increase or decrease has opposing effects on fitness. Organismal trade-offs prevent a single super-species from dominating, making them fundamental in determining community structure and diversity (Tilman, 1990; Kneitel and Chase, 2004) and have been described for microbes, starting with classical work by Pirt (1965). Trade-offs defines diverse ecological strategies that are selected for in different environments and allow coexistence of competitors (Tilman, 1982; Bohannan et al., 2002; Porter and Rice, 2012; Wallenstein and Hall, 2012). Therefore, characterizing trade-offs in microbes should allow us to better understand the mechanisms of community organization. At present, there are a number of trade-offs postulated for diverse microorganisms and for some microbial groups there is enough empirical data to confirm those trade-offs (Litchman et al., 2007; Winter et al., 2010; Edwards et al., 2012; Wallenstein and Hall, 2012). However, many more trade-offs remain not even theoretically derived, much less empirically documented. Most observed and hypothesized trade-offs are pairwise but higher-dimensional trade-offs are possible (Edwards et al., 2011; Shoval et al., 2012) and may provide even more opportunities for coexistence and diversity of strategies.

Here we discuss how resource utilization traits and the potential trade-offs among them can be used to explain patterns of microbial community structure, diversity, temporal dynamics, spatial distribution, and biogeochemical impacts at present and in the future. Obviously, there are many other microbial traits that are important for understanding the structure and dynamics of communities, such as traits determining responses to diverse environmental factors (pH, temperature, pressure), quorum sensing traits, dormancy, etc., but we focus on resource utilization traits because they provide the most direct link between microbial community structure and biogeochemistry and are well described and modeled for many microbes.

We provide examples for both prokaryotic and eukaryotic microbes, phytoplankton in particular, because many relevant phytoplankton traits have been measured, compiled, and used to infer the mechanisms of community organization. Phytoplankton are globally important microbes contributing about half of the Earth’s primary productivity and playing a major role in many biogeochemical cycles, such as carbon, nitrogen, phosphorus, and silica (Field et al., 1998). Recently, much progress has been achieved in applying trait-based approaches to understand the structure and dynamics of phytoplankton communities in both marine and freshwater environments (Edwards et al., 2013b,c). Resource utilization and competition for resources is thought to be a major structuring force in phytoplankton communities (Tilman, 1982), hence, resource-related traits provide crucial information on how individual species respond to environmental conditions, resource supply in particular.

## Resource Acquisition and Utilization Traits and Trade-offs

The classical models that describe resource-dependent growth and utilization in microbes are based on Monod and Michaelis–Menten formulations. According to the Monod equation, the growth rate μ is a saturating function of external resource concentration *R*:

$$\mu =\mu_{\max}\frac{R}{R+K_{s}}\,\;\;\;\;\;\;\left(1\right)$$

where μ_max_ is the maximum growth rate and *K_s_* is a half-saturation constant for growth. The net growth then is μ - m, here *m* is mortality rate (including metabolic losses). The ability to effectively utilize resources in microbiological literature is often calculated as affinity ($\frac{\mu_{\max}}{K_{s}}$; Healey, 1980; Button, 1994; Nedwell and Rutter, 1994). Another measure of competitive ability *R^∗^*, the break even nutrient concentration at which growth equals mortality can be determined (Tilman, 1982):

$$R*=\frac{mK_{s}}{\mu_{\max}-m}\,\;\;\;\;\;\;\;\;\;\;\;\;\left(2\right)$$

which incorporates the effect of loss processes. Species competitive ability increases with decreasing *R^∗^* (Tilman, 1982). According to resource competition theory, the outcome of competition for resources can be predicted from comparing *R^∗^*s of competitors: a species with the lowest *R^∗^* wins competition because it can reduce resource concentration to the levels at which other species cannot survive (Tilman, 1982). The beauty of this approach is that outcomes of competition can be predicted from the monoculture data alone, in contrast to the Lotka–Volterra approach, where competition coefficients need to be measured for every interacting pair of species.

The Monod equation for growth works reasonably well describing growth in constant environments but when resources fluctuate, the model often cannot capture population dynamics adequately (Grover, 1988; Grover, 1991a,c). Droop (Droop, 1973, 1983) formulated an alternative expression for growth rate μ is a function of internal resource concentration or quota *Q*:

$$\mu =\mu_{\infty }\left(1-\frac{Q_{\min}}{Q}\right)\,\;\;\;\;\;\;\;\;\;\left(3\right)$$

where μ_∞_ is the theoretical growth rate at infinite quota and Q_min_ is the minimum quota (internal concentration at which μ = 0). Resource uptake rate *V* is a function of external resource concentration *R*:

$$V=V_{\max}\frac{R}{R+K_{m}}\,\;\;\;\;\;\;\;\;\;\;\left(4\right)$$

where V _max_ is the maximum rate of uptake and K_m_ is the half-saturation constant for uptake. Note that this constant is different from the half-saturation constant for growth in Eq. 1 (it is usually lower). Often, the uptake rate also slows when the internal resource concentration is increasing, so that *V* may also depends on *Q* (Grover, 1991c): equation

$$V=V_{\max}\frac{R}{R+K_{m}}\frac{Q_{\max}-Q}{Q_{\max}\,-Q_{\min}}\,\;\;\;\;\;\;\;\;\;\left(4a\right)$$

where *Q_max_* is the maximum internal resource concentration. Ignoring maximum quotas, if the net growth is μ-m, we can derive the expression for *R^∗^,* a measure of competitive ability at equilibrium (uptake is described by Equation 4):

$$R*=\frac{K_{m}\mu_{\infty }Q_{\min}m\,}{V_{\max}(\mu_{\infty }-m)-\mu_{\infty }Q_{\min}m}\,\;\;\;\;\;\;\;\;\left(5\right)$$

As with the Monod equation, deriving *R^∗^* is possible from the monoculture data for a given mortality but more trait information is needed (three traits instead of two, plus mortality).

### Trait Plasticity and Evolution

Traits characterizing resource uptake and utilization in a single genotype may change depending on growth history and environmental conditions, exhibiting phenotypic plasticity, also frequently called acclimation. For example, the maximum uptake rate, half-saturation constant, and affinity are functions of temperature (discussed in more detail below; Aksnes and Egge, 1991; Reay et al., 1999). Temperature dependence appears to differ for different resources: nitrate utilization by bacteria depended on temperature much more than ammonium utilization (Reay et al., 1999). There are not many systematic studies investigating phenotypic plasticity related to resource utilization in microbes, perhaps with the exception of *Escherichia coli* and some phytoplankton species (e.g., Rhee, 1974; Rhee and Gotham, 1981a,b; Wynne and Rhee, 1986; Wirtz, 2002).

In addition to phenotypic plasticity, resource uptake and utilization traits may evolve in response to diverse selection pressures, resource limitation in particular. Low resource concentrations may lead to an evolution of more efficient resource utilization: *E.coli* evolved more efficient glucose uptake kinetics under glucose-limited conditions (Helling et al., 1987). Similarly, long-term phosphorus limitation in an *E. coli* strain made it a superior competitor for P compared to ancestral strain and to another evolved strain, but, interestingly, decreased its ability to use organic carbon. Specific mutations related to organic carbon metabolism were identified as contributing to enhanced fitness under P limitation (Behrends et al., 2014). Resource utilization traits in microbes can also evolve in the presence of a competitor and the degree of evolutionary response may be asymmetric between competitors (Pekkonen et al., 2013).

### Trade-offs Within and between Resources

Various constraints (energy, resources) on investing into different physiological and ecological functions inevitably lead to trade-offs. Trade-offs between the acquisition of different resources are often postulated and shown to be among the major trade-offs contributing to species coexistence (Tilman, 1982, 1990). They have been demonstrated empirically for phytoplankton and some bacteria (Tilman et al., 1982; Edwards et al., 2011; Behrends et al., 2014). Mechanistic reasons for such trade-offs could be the limited cell surface that could be dedicated to an uptake of a particular resource, given the specificity of uptake sites, or a limited cell volume (in small cells) to accommodate the cellular machinery to process different resources (Aksnes and Egge, 1991; Litchman et al., 2007; Dao, 2013).

When considering the uptake and utilization of a single resource, a negative relationship between the maximum rate of uptake and uptake affinity representing a trade-off between resource utilization under low and high, (or fluctuating) resources has been hypothesized and described for phytoplankton and bacteria (Healey, 1980; Button et al., 2004). There may also be a trade-off between rapid growth and resource storage capacity (high *Q_max_*, Eq. 4a), often related to cell size (smaller cells grow faster but have lower storage capacity [Grover, 1991c]). This trade-off may promote coexistence under resource fluctuations, with fast growing species responding first to resource pulses and the high storage species able to maintain growth at a later phase when resource pulse is getting depleted (Litchman et al., 2009).

Because resource uptake of microorganisms strongly depends on cell size, incorporating cell size into models of resource utilization and competition is important, especially because cell size often influences other community interactions (e.g., predator-prey) and biogeochemical cycles (e.g., carbon sequestration in the ocean) and can be considered a “master trait” (Litchman and Klausmeier, 2008). Cell size can be incorporated into models either by making standard resource utilization traits scale allometrically with cell size or more mechanistically, deriving resource utilization traits from first principles (Armstrong, 2008; Litchman et al., 2009; Fiksen et al., 2013). There are many physical, physiological constraints, and evolutionary pressures that control cell size of microbes (Koch, 1996; Raven, 1998) and thus affect their resource utilization traits.

Good competitive ability for a resource (e.g., nutrient) may also trade-off with other traits, such as susceptibility to viral attacks, due to viruses entering cells through nutrient uptake sites (Jessup and Bohannan, 2008; Menge and Weitz, 2009). A trade-off between resource competitive abilities and sensitivity to antibiotics and detergents has been demonstrated for *E. coli* and a similar mechanism (high membrane permeability) proposed (Phan and Ferenci, 2013).

### Efficiency and Growth Rate Trade-off

Heterotrophic microbes are thought to experience a fundamental thermodynamic trade-off between the rate and yield of ATP production in the breakdown of organic compounds (Pfeiffer et al., 2001). Alternative metabolic pathways allow a plastic response such that yield is higher under low resource availability and production rate is higher under high resource availability (Molenaar et al., 2009). A related trade-off in microbes is between metabolic efficiency and cost (of enzyme production) that can slow growth rate (Flamholz et al., 2013). The original Pirt (1965) model incorporates such a trade-off: it has two parameters, the maintenance energy requirement, and the maximum yield (yield when growth is highest). In terms of the rate-yield trade-off, species with a higher growth rate should have a cost of higher maintenance energy and/or lower maximum yield.

The growth-efficiency trade-off presumably produces different strategies across genotypes and species, and there is partial support for such an evolutionary trade-off in the long-term evolution experiments on *E. coli* (Novak et al., 2006). Changing environmental conditions, such as the rate, variability, and spatial structure of resource supply, should cause shifts in community structure by changing the optimal strategy (Pfeiffer et al., 2001), and in a fluctuating environment multiple strategies could coexist.

### Trade-off Dimensionality

Considering the importance of trade-offs for explaining trait evolution, community structure, and maintenance of diversity, it is surprising that we do not have a better understanding of the important trade-offs for any kind of organism, including microbes. A significant part of the challenge is quantifying the relevant traits for a large number of organisms, but an additional challenge is the high-dimensional nature of ecological interactions. For example, phytoplankton require light and many nutrients, which occur in a variety of molecular forms; under fluctuating resource supply they may specialize on rapid vs. efficient use of resources (gleaner-opportunist trade-off); and they are subject to attack by taxonomically diverse enemies. Therefore, quantifying the cost of high performance for a particular trait may only be possible when multiple other traits are measured.

In a comparative analysis of phytoplankton nutrient utilization traits, we found evidence for a three-way trade-off: competitive ability for nitrate and phosphate (as quantified by uptake affinity relative to the subsistence quota) tend to be negatively correlated for a given cell size, while competitive ability for both nutrients declines as cell size increases (Edwards et al., 2011). Furthermore, the trade-off in N vs. P competition was primarily evident in freshwater species, which likely experience a wider range of N:P supply than marine species (Sterner and Elser, 2002). In a second study we considered strategies for P competition when the supply varies over time. We found that competitive ability under chronically low supply (scaled uptake affinity), maximum growth rate under saturating supply, and P storage capacity exhibit a three-way trade-off (Edwards et al., 2013a). This means that an increase in any one of these traits tends to diminish the other two traits, but these relationships are only detectable in a multivariate analysis. A model shows that this multidimensional trade-off promotes the coexistence of multiple strategies, as well as shifting community structure as a function of the P supply regime.

These results represent tantalizing glimpses into multivariate trait correlations and high-dimensional trade-offs. Quantifying these trait relationships will permit tighter theory-data linkages that should enhance prediction of community structure and ecosystem processes from environmental conditions. Developing this approach will require many trait measurements on many species, and an increased focus on higher trophic levels and pathogens to complement our understanding of resource utilization traits.

### Trade-off Hierarchy

**FIGURE 1:**
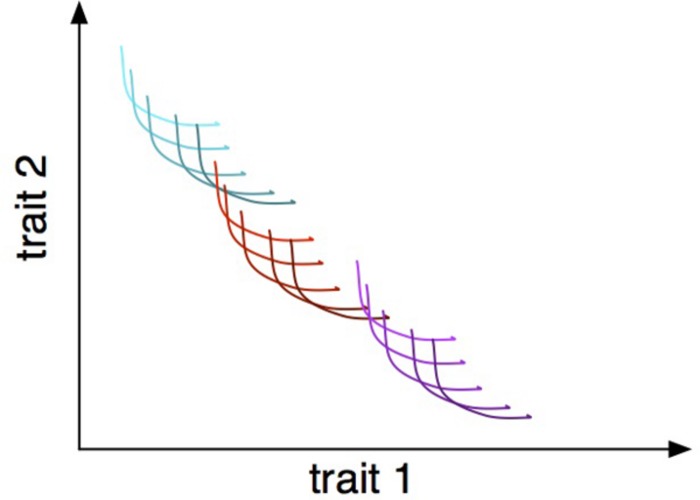
Trade-offs between two traits at three hierarchical levels: within-individual phenotypic plasticity, represented as individual curves; within-species genetic variation, represented as differently shaded curves; and within-community interspecific variation, represented as different colored families of curves

The notion of trade-offs applies to different levels of biological organization. Resource utilization trade-offs in microbes occur at the subcellular level, when different metabolic networks are compared, at the level of individual cells, within a species across genotypes and, finally, across multiple species and higher taxonomic groups and even communities (**Figure 1**). For example, a rate-efficiency trade-off is observed in metabolic networks, where networks are either high yield but slower rate or *vice versa* (Molenaar et al., 2009). The same trade-off is reported for individual species of bacteria (Maharjan et al., 2007; Frank, 2010), across species (Flamholz et al., 2013) and even among communities (Lipson et al., 2009). Identifying and connecting trade-offs at different levels is a formidable challenge but should provide a link across these levels and across disciplines, from systems biology to population and community to ecosystem ecology. Trait diversity at different hierarchical levels will have their own characteristic time scales, which will affect the speed at which communities respond to changing environmental conditions. For example, phenotypic plasticity may maintain ecosystem functioning but slow down evolutionary adaptation by decreasing the fitness differential between genotypes.

### Trade-offs, Coexistence, and Community Diversity

**FIGURE 2:**
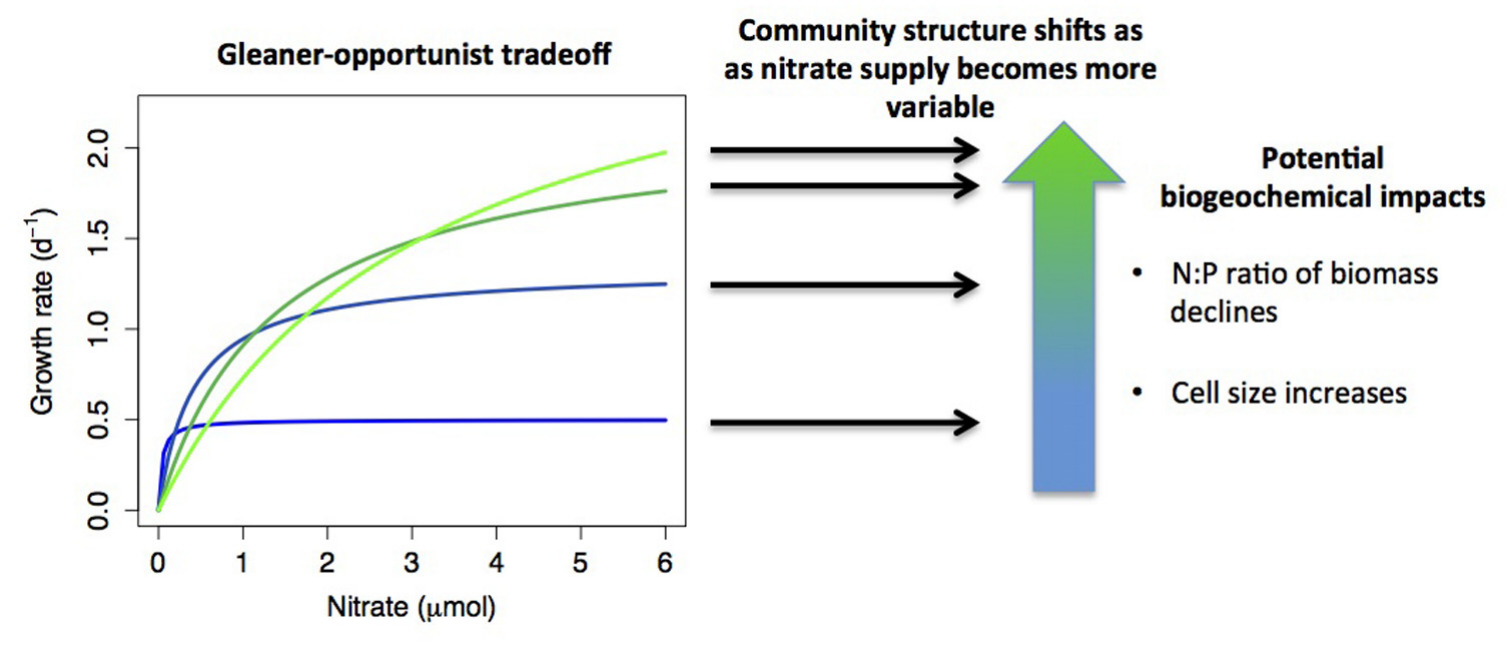
A hypothetical example of how resource acquisition trade-offs interact with environmental conditions to determine community structure, with biogeochemical consequences. Monod growth curves are plotted for four species that exhibit an ‘opportunist-gleaner’ trade-off, where high maximum growth rate comes at a cost of reduced affinity. If nutrient supply shifts from relatively constant to highly variable, this shifts the relative fitness of the different strategies, with different species dominating under different conditions. Shifts in community composition may have biogeochemical impacts, such as reduced biomass N:P when opportunists dominate (growth rate hypothesis), or increased phytoplankton cell size (smaller cells tend to have higher affinity). A further possibility is that multiple strategies may coexist under variable nutrient supply, which is not depicted in the diagram.

Pairwise and higher-dimensional trade-offs in resource utilization traits can lead to coexistence of many competitors and thus generate significant microbial diversity. Fluctuating resource conditions, spatial heterogeneity, predators, and parasites (e.g., phages and grazers) select different species based on a diverse set of trade-offs and can promote coexistence (**Figure 2**). Moreover, resource utilization trade-offs can lead to an evolutionary diversification of social strategies, depending on environmental conditions (Kreft and Bonhoeffer, 2005). The knowledge of trade-offs and other relationships among traits can also help infer missing traits for individual microbes (Edwards et al., 2011). As we do not have a good understanding of the nature, dimensionality, or the shapes of many potential trade-offs, a better characterization of such trade-offs is important for getting at the mechanisms of community assembly and diversity.

## Using Resource Acquisition Traits to explain Temporal and Spatial Patterns in Natural Microbial Communities

One of the main goals of trait-based approaches in community ecology is to explain the composition and dynamics of communities. In terrestrial plant ecology, many traits used to explain community patterns are measured on individuals *in situ* and can be related to community composition under given environmental conditions. For example, several leaf traits explain plant community composition changes along temperature and precipitation gradients (Wright et al., 2004). In microbes, including phytoplankton, measuring traits of individuals *in situ* is virtually impossible aside from cell size. The information about resource utilization traits mostly comes from laboratory measurements on cultured microbial populations (Button, 1985; Edwards et al., 2012). A comparison of these measured traits reveals significant variation among species, thereby supporting the importance of community composition for the total resource dynamics and the community composition effects on resource stoichiometry (Grover, 1989; Arrigo et al., 1999; Edwards et al., 2012).

Can we use these lab-measured traits to explain and predict the responses of microbial populations to environmental drivers, resource variation in particular, in real ecosystems? How representative are these trait values of the microbial utilization differences *in situ*? If they are, then culture experiments to measure traits on more microbial populations would be valuable for predicting community structure and functioning in natural conditions. Hence, we would need to revive culture-based studies, combined with the “omic” approaches and develop novel culturing methods to be able to measure traits of the previously unculturable microbes (Puspita et al., 2012; Editorial, 2013).

As it is unrealistic to expect that we can measure traits of most microbes from natural communities, given the incredible microbial diversity and culturing difficulties, could we focus on traits of ecologically/biogeochemically important species to capture major patterns in community structure and dynamics? Some studies indicate that a relatively small fraction of species in microbial communities may be responsible for the majority of biogeochemical functions (Gobet et al., 2012). Consequently, measuring resource utilization traits of such a subset of species may prove sufficient.

We have found that lab-measured phytoplankton traits can be used successfully to explain spatial and temporal variation in community structure in lakes and the ocean. Because phytoplankton are diverse but few species have been studied in culture, we characterized community structure along environmental gradients by quantifying the differential responses of individual species. For example, we predicted that a species with a higher competitive ability for nitrate (higher scaled uptake affinity) should increase in relative abundance as nitrate becomes scarce. This prediction can be tested with a statistical model that quantifies the slope of response to environmental predictors, for a group of species, and compares species-specific response slopes to those species’ traits. We applied this approach to the English Channel L4 phytoplankton time series (Widdicombe et al., 2010; http://www.westernchannelobservatory.org.uk/). We found that (1) better nitrogen competitors respond more positively to decreased nitrate; (2) species with higher light-limited growth rate respond more positively to decreased mixed-layer irradiance; (3) species with higher maximum growth rate response more positively to combined light and nitrogen availability (i.e., ‘bloom’ conditions; Edwards et al., 2013b).

We applied the same approach to phytoplankton community variation across North American lakes (Edwards et al., 2013c). In this case we again found that species with higher light-limited growth respond more positively as irradiance decreases, and that species with higher maximum growth rate respond more positively to combined light and phosphorus availability. For both of these analyses we compiled lab-measured traits from many prior studies; these studies used different methods, and they measured traits on isolates that were not collected from the same sites where the survey data was collected. We nonetheless found results consistent with *a priori* predictions, which is a strong indication that resource utilization traits are effective predictors of community structure in space and time.

Another approach to determine the role of resource utilization traits and resource-based interactions in microbial communities is assembling communities of members with well-defined resource utilization traits and subsequently characterizing species and community dynamics. Such approach has long been applied successfully to investigate resource competition in phytoplankton (Tilman, 1982; Grover, 1988, 1991b) and, more recently, syntrophy in bacteria, including the multispecies communities (Mee et al., 2014). “Omic” approaches can help define resource requirements, metabolic strategies and ecological niches for diverse microbes (Muller et al., 2014) and, thus, facilitate assembling synthetic communities with known traits.

### Genomic Signatures of Resource Utilization Traits

Because it is impossible to measure all these traits for a majority of microbes, even with better culturing techniques, finding alternative ways to infer trait values would be extremely helpful. With the rise of various methods to obtain genomic, transcriptomic, and other “omic” data for individual species and communities, it is tempting to explore the links between genomes and the physiological, including the resource utilization, traits. Comparative approaches, where genomes of many species or strains are compared to the corresponding values of their physiological traits should be particularly illuminating. For example, relating the number of nitrate transporter genes (copies) to measured nitrate uptake affinity in different species could determine whether such relationship exists, how significant it is and whether it could be used to predict uptake affinities. At present, however, establishing such quantitative relationships may still be out of reach because we either lack annotated genome information or do not have traits measured on a sufficient number of species. The presence of the different affinity uptake systems, their complexity and numerous genes encoding them further complicate potential inferences. Interestingly, the same phenotypic traits can be achieved by very different metabolic profiles, so that there may be higher redundancy at the subcellular level than at the level of a phenotype (Maharjan et al., 2007). Integrating more complete genomic and metabolic network information into quantitative indices and relating them to resource utilization traits through multivariate models could yield predictive relationships.

## Using Resource Utilization Traits to Quantify Biogeochemical Impacts

### Traits and Stoichiometry

Resource utilization traits of individual species or genotypes, being simultaneously response and effect traits, when scaled up to the whole community, determine the microbial impacts on biogeochemical cycles. Uptake of inorganic nutrients by microorganisms, for example, affects ambient nutrient concentrations and, because different groups of microorganisms have different nutrient requirements, microbial community composition also determines stoichiometric ratios of major elements in nature (Arrigo et al., 1999; Elser et al., 2000; Lenton and Klausmeier, 2007; Kaiser et al., 2014). For example, in the Southern Ocean phytoplankton communities dominated by diatoms assimilate CO_2_ and nitrogen at much lower ratios to phosphorus than communities dominated by the prymnesiophyte *Phaeocystis antarctica* (Arrigo et al., 1999). Different requirements for major nutrients among microbial groups may manifest physiological trade-offs in investment and define contrasting ecological strategies (Klausmeier et al., 2004). These, in turn, modify elemental stoichiometry of microbial communities and the environment. For example, according to the growth rate hypothesis, fast growing organisms (e.g., *r*-strategists, opportunists) invest more phosphorus into ribosomes and because ribosomes are relatively P-rich, that results in high P concentration and, consequently, low C:P and N:P ratios in biomass (Elser et al., 1996; Makino et al., 2003). In contrast, slow growing organisms may invest more into resource acquisition machinery (nitrogen-rich proteins), thus increasing their competitive abilities (*K*-strategists, gleaners) and, consequently, having high N:C and N:P biomass ratios. Environmental conditions that select for fast growth (e.g., high resource environments or fluctuating conditions) would then favor microorganisms with low N:P and C:P ratios and alter biomass stoichiometry (Klausmeier et al., 2004). These groups would likely acquire more P from the environment, thus immediately decreasing P availability for competitors. On longer time scales, however, the dominance by the low N:P ratio groups and subsequent recycling of their biomass would lead to low N:P and C:P ratios, matching the dominant phytoplankton group stoichiometry (Klausmeier et al., 2004; Arrigo, 2005). Changing selection regimes favoring microbial groups with certain N:P ratios are hypothesized as a mechanism for the non-Redfield ratios in the ocean (Klausmeier et al., 2004; Arrigo, 2005).

### Trait-Based Biogeochemical Models

To estimate the effects of microbial communities on biogeochemical cycles, individual resource utilization rates need to be scaled up to community level by taking into account biomass of contributing microbes. Such approaches are being commonly used for terrestrial plants and marine phytoplankton to estimate primary productivity and biogeochemical cycling at large scales (Lavorel and Garnier, 2002; Moore et al., 2002; Pavlick et al., 2013) and are starting to be applied to other microbes (Allison, 2012). There has been a growing realization for both the macroorganismic and microbial systems that to adequately represent biogeochemical processes under dynamic environmental conditions, biogeochemical models need to explicitly include community diversity, especially of organisms with different biogeochemical signatures. For example, ocean biogeochemical models progressed over the years from considering just bulk phytoplankton to resolving major functional biogeochemically distinct groups because changes in abundances of these groups significantly impact elemental balance and biogeochemical cycling. Adding more microbial groups to models presents significant challenges for parameterization, computational manageability, and mechanistic interpretability of results. Using trait-based approaches, where the focus is on traits, not on species/groups *per se* and where both response and effect traits are included, allows reducing model complexity while preserving biogeochemical relevance (Bruggeman and Kooijman, 2007; Follows and Dutkiewicz, 2011). Incorporating relevant trade-offs among traits allows an even further reduction of complexity, reducing the number of traits that need to be considered explicitly (Litchman and Klausmeier, 2008; Merico et al., 2009). Another advantage of trait-based community models is that it is easier to incorporate potential trait evolution in response to selection by the changing environment, compared to species-based models (Litchman et al., 2009; Merico et al., 2009). The successes of trait-based biogeochemical models that explicitly incorporate community diversity (i.e., a much better representation of biogeochemical transformations compared to bulk models) underscore the importance of knowing resource utilization traits of key microbial players and the limited predictive utility of measuring only the bulk biogeochemical traits.

### Global Change Effects

Microbes are sensitive to global environmental change, including rising CO_2_ levels, temperatures, increased nitrogen deposition, and other alterations of nutrient levels. Moreover, sensitivity differs among groups and individual species, thus causing changes in community composition and functioning. Differential temperature sensitivity across microbial groups and species is well documented (Ratkowsky et al., 1982; Corkrey et al., 2012; Thomas et al., 2012). Consequently, warming can stimulate or inhibit microbial groups that also differ in their resource utilization traits which would alter the distributions of different elements in the microbial biomass and their availability in the environment (Hoffmann et al., 2012). Therefore, rising temperatures can affect biogeochemistry indirectly, through changing microbial community composition.

Temperature also affects biogeochemical processes directly, by modifying the microbial kinetics of resource uptake and utilization according to thermodynamic and catalysis laws (Winkle, 1999). Major resource utilization traits depend on temperature: both the maximum rate of resource (nutrient) uptake rate and half-saturation constant for uptake are predicted to increase with temperature (Aksnes and Egge, 1991). However, *V*_max_ is predicted to increase faster than *K_s_* with temperature (Aksnes and Egge, 1991); hence, rising temperatures may lead to an increase in uptake affinity (*V*_max_/*K_m_*) and, consequently, more efficient resource utilization and lower residual resource concentration in the environment (lower *R^∗^*). Similarly, for a Monod-type growth description, the resource-dependent growth affinity (μ_max_/K_s_) is low at low temperatures and increases when the temperatures approach optimum temperatures for growth (Reay et al., 1999). Less efficient resource utilization under suboptimal temperatures was shown experimentally and in the field, for both bacteria and microalgae (Nedwell and Rutter, 1994; Reay et al., 1999; Pomeroy and Wiebe, 2001).

**FIGURE 3:**
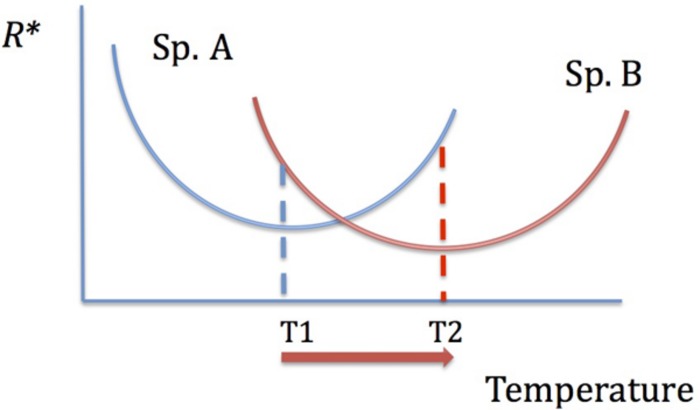
The dependence of resource competitive ability on temperature. The species with the lowest *R^∗^* is the best competitor. With increasing temperature, there is a shift in competitive abilities: species A is a better competitor at *T1* and species B is a better competitor at *T2*.

It is likely, however, that an increase in the uptake or growth affinity with increasing temperature would occur only up to the optimum temperature for a given uptake enzyme(s) activity or for growth, and would decline after that. Indeed, the residual nutrient concentrations were shown to increase past the optimum temperature for growth in bacterial cultures (Reay et al., 1999), suggesting less efficient resource utilization. Therefore, determining the temperature dependence of growth in different bacterial species would allow us to predict whether increasing temperatures would lead to a decreased or increased resource utilization by those species. Temperature, thus, mediates resource competition: species and groups that have their temperature optima most closely matching future temperatures will perform their best in resource competition and resource utilization (**Figure 3**). Note, however, that those species may still be inferior competitors compared to the overall best competitors. Evolutionary adaptation to temperature, well documented in microbes (Bennett and Lenski, 1993; Mongold et al., 1996), can further alter competitive hierarchies, especially if microbial species have different adaptive potential.

The changing availability of resources (e.g., inorganic nutrients, organic substrates) is another consequence of anthropogenic environmental change. Increased atmospheric nitrogen deposition, eutrophication, and “brownification” (increased leaching of organic carbon from soils into aquatic ecosystems) are among the many examples of resource alterations. Altered regimes of resource supply change microbial community composition by selecting species/groups with particular microbial resource utilization traits. For example, high or fluctuating inputs of resources may select for the fast growing or the high maximum uptake rate or the high storage capacity species (Grover, 1991c; Litchman et al., 2009). The period of fluctuations also matters for community composition and dynamics: bacterial communities that experienced different frequencies of resource (protein) pulses were highly dissimilar across pulse regimes (Carrero-Colon et al., 2006). Moreover, growth rates of isolates from the longest pulse period (14 days) were lower than for other pulse regimes. Interestingly, despite the dissimilarity in community composition, at least some aspects of community functioning (e.g., aminopeptidase activity) were similar, suggesting functional redundancy (Carrero-Colon et al., 2006). Global environmental change is predicted to modify not only the means of environmental variables, including resources, but temporal patterns (variance) as well (e.g., Chou et al., 2011; Seneviratne et al., 2014) and that can have a profound effect on microbial communities, in part because of the differences in species resource utilization traits and numerous trade-offs.

## Concluding Remarks

Classical mathematical descriptions of resource utilization and competition in microbes, as well as novel models including more realism provide a firm foundation for exploring the principles of microbial community organization at present and under future environmental conditions. Parameters of these models are key functional traits of microbes that determine their community structure, dynamics, and biogeochemical impacts. Characterizing these traits for many diverse microbial species through lab measurements using novel culturing techniques and “omic” approaches should provide rich data for model parameterizations. Trade-offs at different levels of biological organization and of different dimensionality are essential for predicting microbial community assembly and evolution and should be better characterized. Together, trait and trade-off information and realistically parameterized mathematical models, will be instrumental in increasing our mechanistic understanding of how natural ecosystems function.
